# Towards the direct detection of viral materials at the surface of protective face masks via infrared spectroscopy

**DOI:** 10.1038/s41598-022-06335-z

**Published:** 2022-02-10

**Authors:** Vanessa Schorer, Julian Haas, Robert Stach, Vjekoslav Kokoric, Rüdiger Groß, Jan Muench, Tim Hummel, Harald Sobek, Jan Mennig, Boris Mizaikoff

**Affiliations:** 1 Institute of Analytical and Bioanalytical Chemistry, Ulm University, Albert-Einstein-Allee 11, 89081 Ulm, Germany; 2 Hahn-Schickard, Sedanstraße 14, 89077 Ulm, Germany; 3 Institute of Molecular Virology, Ulm University Medical Center, Meyerhofstr. 1, 89081 Ulm, Germany; 4 Labor Dr. Merk & Kollegen GmbH, Beim Braunland 1, 88416 Ochsenhausen, Germany; 5 Present Address: Boehringer Ingelheim Therapeutics GmbH, Beim Braunland 1, 88416 Ochsenhausen, Germany

**Keywords:** Mid-infrared photonics, SARS-CoV-2

## Abstract

The ongoing COVID-19 pandemic represents a considerable risk for the general public and especially for health care workers. To avoid an overloading of the health care system and to control transmission chains, the development of rapid and cost-effective techniques allowing for the reliable diagnosis of individuals with acute respiratory infections are crucial. Uniquely, the present study focuses on the development of a direct face mask sampling approach, as worn (i.e., used) disposable face masks contain exogenous environmental constituents, as well as endogenously exhaled breath aerosols. Optical techniques—and specifically infrared (IR) molecular spectroscopic techniques—are promising tools for direct virus detection at the surface of such masks. In the present study, a rapid and non-destructive approach for monitoring exposure scenarios via medical face masks using attenuated total reflection infrared spectroscopy is presented. Complementarily, IR external reflection spectroscopy was evaluated in comparison for rapid mask analysis. The utility of a face mask-based sampling approach was demonstrated by differentiating water, proteins, and virus-like particles sampled onto the mask. Data analysis using multivariate statistical algorithms enabled unambiguously classifying spectral signatures of individual components and biospecies. This approach has the potential to be extended towards the rapid detection of SARS-CoV-2—as shown herein for the example of virus-like particles which are morphologically equivalent to authentic virus—without any additional sample preparation or elaborate testing equipment at laboratory facilities. Therefore, this strategy may be implemented as a routine large-scale monitoring routine, e.g., at health care institutions, nursing homes, etc. ensuring the health and safety of medical personnel.

## Introduction

For over a year now, the Covid-19 pandemic has been affecting the daily lives of people around the world. In March 2020 the World Health Organization (WHO) has declared the outbreak of the severe acute respiratory syndrome coronavirus type 2 (SARS-CoV-2) a global pandemic^1^. Since then, millions of people were infected and nearly all countries fight against the virus by slowing the spread of the virus, and thus, avoiding overwhelmed health systems. These measures involve strategies to detect and stop known chains of transmission. Therefore, the rapid, reliable, and direct detection of SARS-CoV-2 is of particular importance for mitigating infection spreading. Evidently, there is a distinct demand for testing/screening methods identifying individuals with acute respiratory infections, or that have been exposed to respiratory viruses. To date, the gold standard for clinical diagnosis are assays based on detection of viral RNA, mainly reverse-transcriptase polymerase chain reaction (RT-PCR) based methods^2^. Such nucleic acid tests provide high sensitivity and specificity, but also have several drawbacks. The workflow for RT-PCR tests with a duration of nowadays around 40 min includes several steps, which are in part time-consuming and require a laboratory environment, dedicated equipment, and extensive human labor^3^. The development and approval of rapid antigen tests allows for a more rapid detection of infectious individuals (approx. 15 min), and may advantageously be performed outside dedicated laboratories^4^. However, these tests are mainly based on nasopharyngeal swabs, which still requires adequate sample collection routines, and may cause discomfort of patients^5–10^. In addition, using self-collected anterior nasal and saliva specimens are therefore favorable, as the risk for healthcare workers is minimized due to maintaining physical distancing^5,7,10^. Saliva collection is usually conducted via a cotton pad device, i.e., ‘lollipop-technique’ or by spitting into a sterile tube^6,9,10^. The use of self-collected saliva samples is certainly a convenient alternative for swab-based molecular tests, yet, still requires dedicated testing equipment, i.e., cups and solvents^9,10^. To address the direct need for increased routine testing and large-scale monitoring, the present study focus on direct optical read-out techniques, and specifically mid-infrared (MIR) spectroscopy operating in the 3–12 µm spectral regime, which holds promise for the development of a rapid and non-invasive SARS-CoV-2 detection strategy. Herein, we demonstrated an approach to better understand and potentially prevent or reduce exposure to viral respiratory infections such as SARS-CoV-2.

Intriguingly, molecular exposure information is being produced worldwide but has been widely neglected to date. As the pandemic spreads, surgical and/or protective masks are increasingly being worn to prevent infection. Precisely these protective masks and the contaminants filtered/trapped therein including viruses present significant potential for virus detection via preconcentration at the surface of the mask material. Surgical masks are typically made of non-woven, i.e., blown or spun polypropylene fabric, and typically comprise three layers^11–13^. Few analyses of masks have been performed focusing on the evaluation of exhaled breath aerosols (EBA)^14,15^. However, most applied analytical techniques require extensive analytical equipment and labor. Hu et al. presented an approach, which was based on direct analysis via real-time mass spectrometry (DART-MS). In this study, a solid phase microextraction (SPME) fiber was inserted into a face mask for collecting EBA. While this essentially represents a non-invasive approach for characterization human exhaled breath, still additional sampling via SPME and analysis using DART-MS is needed^16^. Analytical procedures classifying surgical face masks without any sample preparation remain rare^16–19^.

Staff in hospitals or clinics, nursing homes and paramedics are subject to a significant risk of infection or contagion demanding for the particularly rapid and reliable detection of SARS-CoV-2, or other pathogens. Within this study, first promising results of directly using face masks as ‘sampling tool’ in combination with attenuated total reflection (ATR) and external reflection (ER) infrared spectroscopy are shown indicating the potential of this strategy via directly detecting and discriminating model analytes including bovine serum albumin (BSA), SARS-CoV-2 virus-like particles (VLPs), and adeno associated virus type 2 (AAVs) based on chemometric classification of the directly obtained IR spectra.

Treating protective face masks as ‘sampling device’, the detection of SARS-CoV-2 is envisioned in a cost-effective way and without requiring any laboratory facilities. Low-cost automated IR spectrometers combined with the developed multivariate data evaluation approach may be integrated into a fully automated screening routine. Thus, testing in mass screening scenarios may be envisaged similar to ion-mobility spectrometry (IMS) based safety routines implemented at airport security checkpoints.

**Figure 1:**
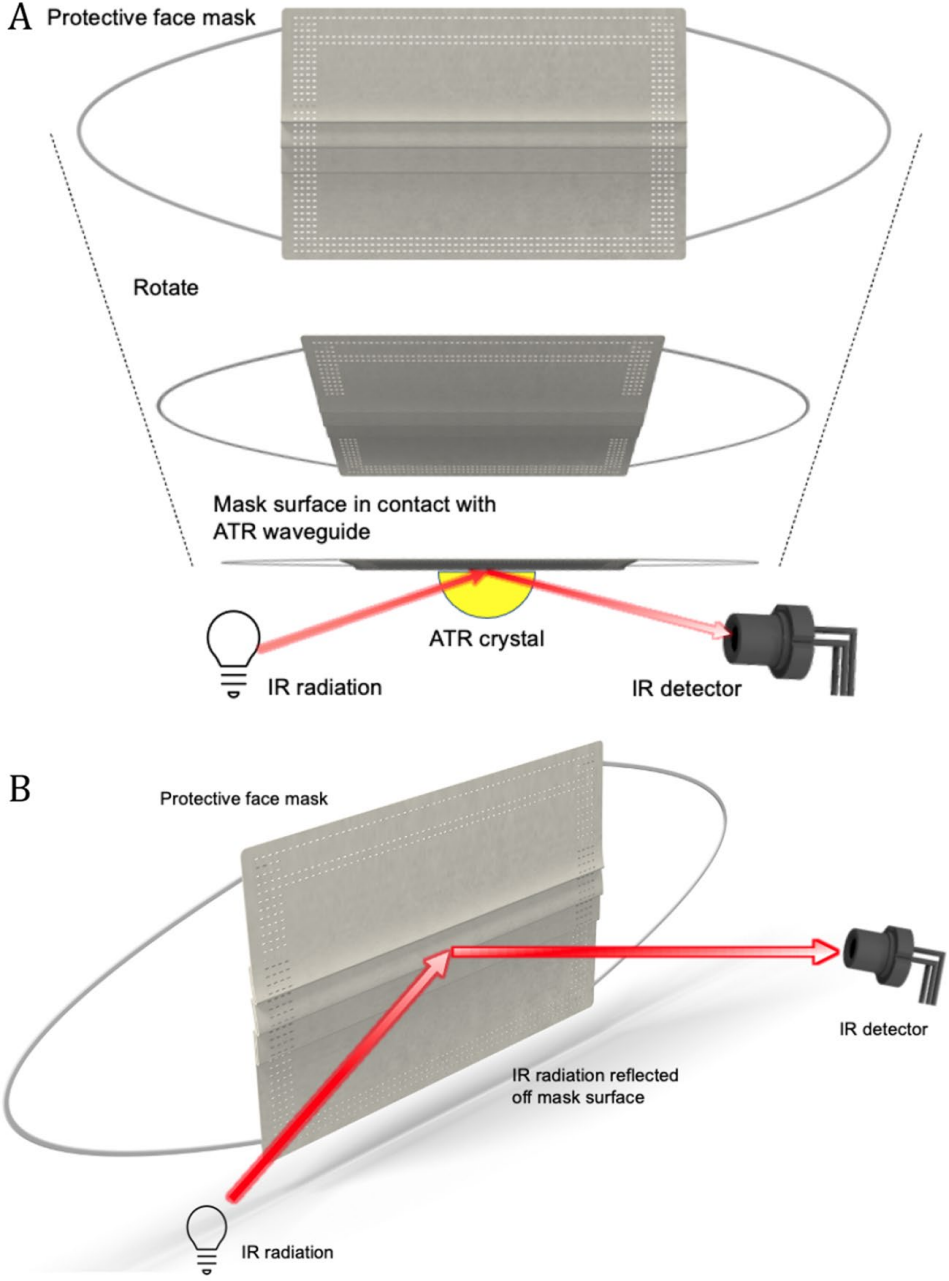
Schematic illustration of (**A**) attenuated total reflection (ATR), and (**B**) external reflection (ER) infrared spectroscopy^20^.

Infrared spectroscopy has matured into one of the most relevant analytical techniques, as it is non-destructive yet provides molecular information on a wide range of organic and inorganic species. The absorption of IR light excites vibrational, rotational, and ro-vibrational transition within molecules depending on the respective phase, i.e., solid, liquid, or gaseous. Thus, obtained spectra enable identifying chemical and structural characteristics essentially ‘fingerprinting’ any molecular species. Likewise, all biomolecules and even entire biological specimen absorb IR radiation providing potential for pinpointing biomaterials including, e.g., virus containing aerosols^21^. While IR spectroscopy is nowadays considered a routine tool for studying protein structure^20^, especially IR-ATR techniques enable the analysis of otherwise opaque samples (Fig. 1A)^22^. Alternatively and if a sufficient signal strength may be obtained, IR–ER—whereby IR radiation is reflected off the external surface of a sample (Fig. 1B)—may be applied, e.g., for direct particle analysis at a filter surface^23–25^. Using multivariate statistics, minute spectral differences may be evaluated rather than focusing on identifying the spectral characteristics of individual molecules, thereby enabling the classification of contaminants at the surface of protective face masks.

The utility of IR spectroscopy in combination with multivariate data analysis derives from the fact that any broadband IR spectrum is composed of a series of characteristic absorption peaks spread along a wavenumber axis ideally suited for analysis via unsupervised methods. Principal components analysis (PCA) followed by principal components regression (PCR) is among the most commonly applied multivariate algorithms projecting variances contained within the data set onto a small number of Eigenvectors (i.e., principal components; PCs) with the aim of reducing dimensionality^26^. Conversely, partial least squares (PLS) regression is a method related to PCA, yet besides fulfilling the criterion that a PC should describe the maximum residual variance simultaneously relates the latent variables to the dependent variables in an optimized way. PLS in combination with linear classification methods yields so-called PLS-based linear discriminant analysis routines (PLS-DA)^27^. While it should be noted that even those rather simple linear multivariate data evaluation routines yielded excellent results in the present case, it is not excluded that currently thriving non-linear algorithms as used in artificial intelligence, machine learning, etc. such as support vector machines (SVMs) and others may be applied as well, albeit usually requiring much more expanded trainings data sets^28^.

A main advantage of multivariate data mining and classification techniques is the fact that the data evaluation/classification may be fully automated, i.e., provides classification data for any end-user without a priori knowledge on the actual measurement routine. While internally a variety of species including molecules, particles and viruses may be detected at the mask surface and could be further analyzed by experts, an end-user may simply be issued a yes/no decision on whether SARS-CoV-2 has been exhaled by an infection person, or whether a healthy person has been exposed to the virus over extended periods during inhalation when wearing a protective face mask as to identify potentially critical exposure scenarios.

The present study aims at a first step towards developing such decision-making models based on surrogate samples and applying IR spectroscopic techniques in combination with PCA and PLS-DA as chemometric data evaluation tools.

## Materials and methods

### Samples and chemicals

For the experiments, conventional surgical face masks were used. The face masks were all fabricated by the same manufacturer (Voin) in line with the European standard EN 14683 for medical use. These masks are three-layered disposable face masks consisting of polypropylene (PP). The two outside layers are non-woven; the intermediate layer was a double layer of melt-blown filter paper. Water (H_2_O), BSA, AAVs and SARS-CoV-2 VLPs were applied to the mask surface by spray or drop casting at the outside/inside emulating deposition after inhalation/exhalation, respectively. Prior to the experiments, crystalline powder of BSA was dissolved in H_2_O (0.5% solution). The SARS-CoV-2 VLPs were generated by transient transfection of plasmids expressing all viral structural proteins in HEK293T cells as previously described^29^, purified by OptiPrep gradient ultracentrifugation, and characterized by nanoparticle tracking analysis. Solutions of NTA-tracked particles of VLP preparation adjusted to 10^10^ particles/ml were added.

AAVs were purchased at Vigene Biosciences (Rockville, USA) as standard reference material.

After the deposition of H_2_O, BSA, AAVs or VLPs, the masks were dried for two hours prior to any measurements. For all experiments ultra-pure water was used (resistivity 18.2 MΩ at 25 °C; Millipore).

### Instrumentation

**Figure 2:**
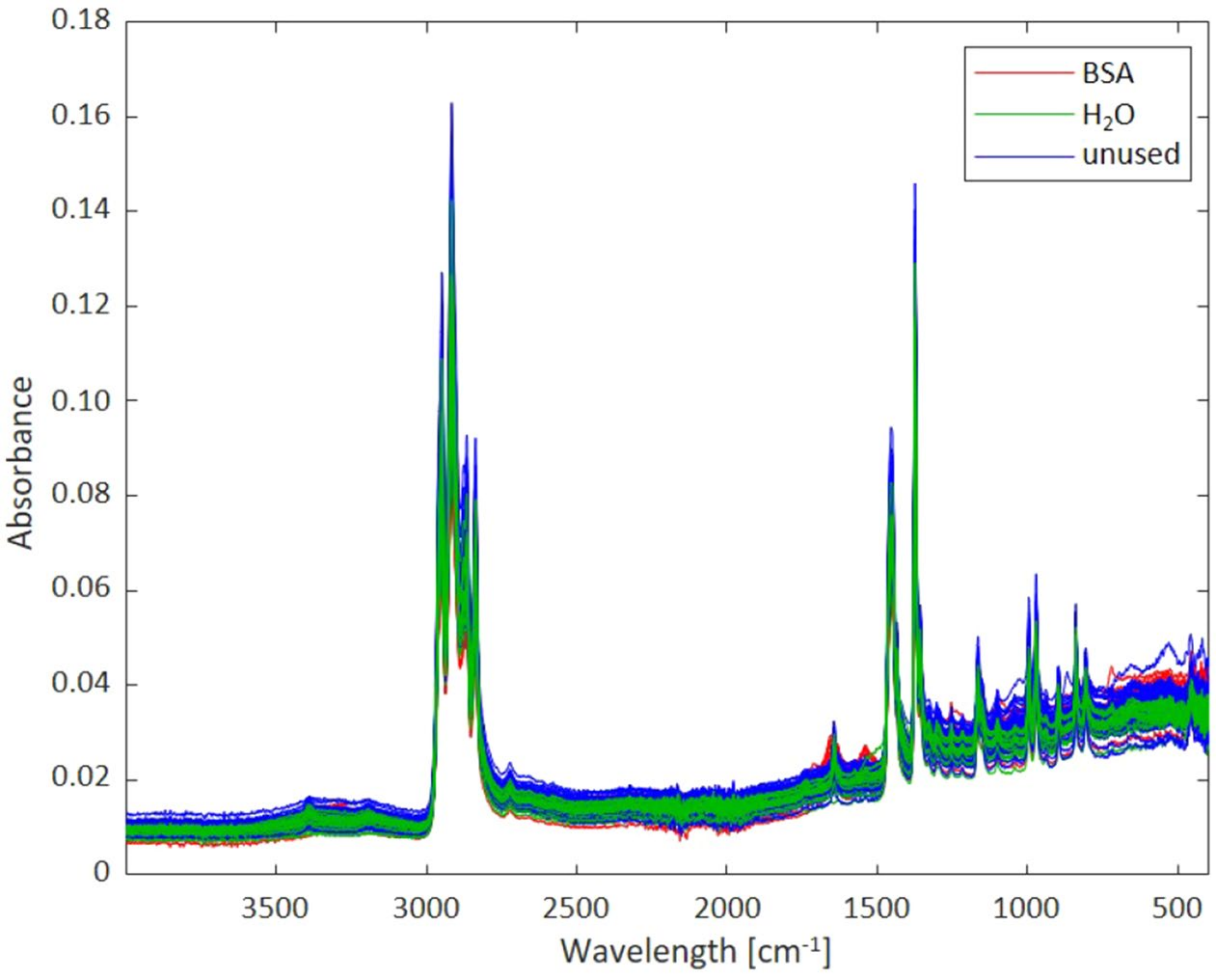
Exemplary characteristic evanescent field absorbance spectra of protective face masks obtained via IR-ATR spectroscopy. Unused masks (blue), masks sprayed with BSA (red), and masks sprayed with water (green).

A portable FT-IR spectrometer (Alpha II HR; Bruker Optics, Ettlingen, Germany) was utilized for all measurements using a single-reflection ATR and an ER assembly, respectively. For ATR spectroscopy, the masks were placed onto the diamond ATR element, and a pressure applicator ensured intimate contact between the mask surface and the crystal ensuring efficient probing via the evanescent field. For ER studies, the samples were pressed evenly towards the aperture of the ER accessory. In both cases, no additional sample preparation was done. For each measurement, IR spectral data in the range of 4000 cm^−1^ to 400 cm^−1^ was collected averaging 128 scans per spectrum at a spectral resolution of 2 cm^−1^. Figure 2 shows exemplary IR-ATR spectra of untreated masks, masks sprayed with H_2_O, and masks sprayed with BSA solutions without any data processing (i.e., raw data), respectively. Five repeat measurements were performed using air as the background spectrum prior to the acquisition of each sample spectrum. For cleaning the ATR crystal after each measurement, isopropyl alcohol (propan-2-ol, IPA) was used.

### Data processing

After spectral data acquisition, pre-processing of the spectra was performed for extracting the relevant information^26,30^. The IR spectra were analyzed using MATLAB (Release 2020a, MathWorks, Natick, USA) with the PLS toolbox 8.7 (Eigenvector Research Inc., Manson, USA). Typically, a variety of pre-processing steps are used to remove unwanted variance (i.e., variance not characteristic for the target analytes) from individual samples and numerically prepare data for modelling^21^. In all models described below, baseline correction, i.e., weighted least squares automatic baseline removal (AWLS, order = 2), and normalization (i.e., 1-norm/standard normal variate) were used to improve the signal-to-noise ratio (SNR) of the data and to adjust the baseline. In addition, applying the 1st derivative Savitzky-Golay filter (filter parameters: order = 2, window: 15 pt) as well as mean centering were used for pre-processing the spectra aiming at a possibly robust model. For proteins, characteristic spectral bands are located in the ‘fingerprint region’ (i.e., 1800 cm^−1^ to 600 cm^−1^), thus, spectral region selection was performed accordingly^22^.

For the established classification models, the number of latent variables (LVs) varies between two and three as selected by evaluating the root mean square error of calibration (RMSEC) and the root mean square error of cross-validation (RMSECV) vs. the number of LVs, which yields an optimum number of LVs yet effectively prevents from modeling noise. Cross-validation (CV) was performed with 10 splits divided by the venetian blinds method using a blind magnitude of 1. The most effective model was selected based on an optimum in sensitivity, specificity, and a minimum CV error by modifying data pre-processing and considering only the fingerprint region (1800–600 cm^−1^), as e.g., features in the high wavenumber regime (3600–2800 cm^−1^) were intensified and contribute little to the overall model performance.

## Results and discussion

As already briefly described, data pre-processing was applied to the raw data prior to modelling for ensuring optimum classification and model performance. All samples were evaluated using PCA and PLS-DA applied separately to both IR-ATR and IR-ER data.

### IR-ATR analysis of protective face masks

In a first set of experiments, masks sprayed with H_2_O and BSA were compared by using PLS-DA. The scores were used to cluster the obtained data. While the obtained RMSEC value provides information on how well the model fits data, the RMSECV determines the ability of the model predicting unknown samples that were not used to build the model. By plotting the RMSEC/RMESCV vs. the number of latent variables, two LVs were selected covering 47.65% of the total variance maximizing the predictive performance of the model and suppressing interferences.

**Figure 3:**
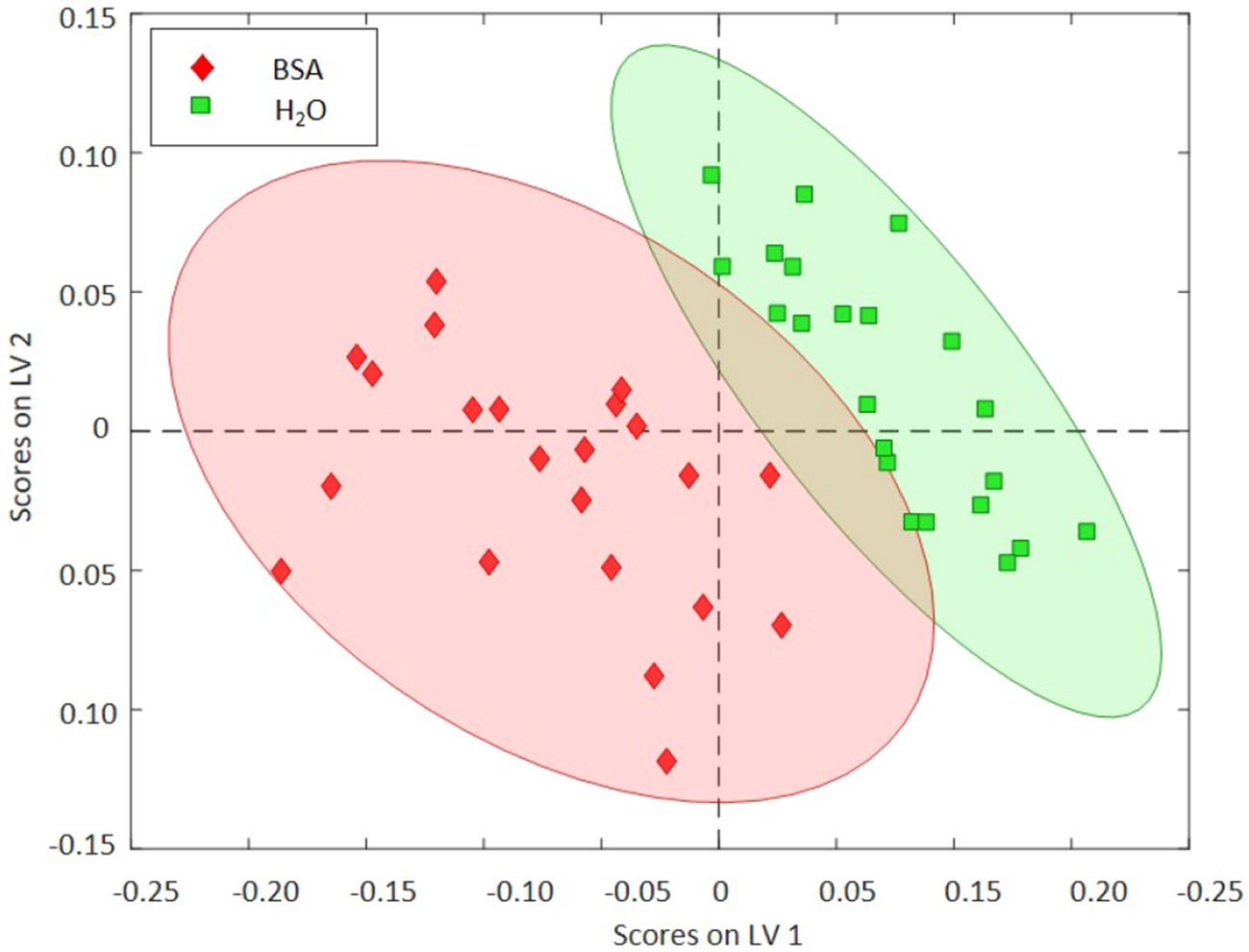
Scores plot of a PLS-DA evaluation at differently treated mask samples (i.e., contaminated with H_2_O and BSA). The two classes are well separated using two LVs based on the IR-ATR data set.

Table 1 summarizes the obtained classification results along with their statistical characterization for all generated PLS-DA models. The coefficient of determination (R^2^) values for calibration and CV verifies a high predictive accuracy of the generated model, as both values are > 0.7. As shown in Fig. 3, the PLS-DA model classifies the masks according to the exemplary analytes H_2_O and BSA. The loadings on LV 1 and LV 2 of this model describe parts of the characteristic H_2_O bending vibration around 1640–1670 cm^−1^. More precisely, a sharp narrow part of the water bending vibration is described, and thus, a structural change of this vibrational mode is indicated. Additionally, the signal at approx. 1390 cm^−1^ increase in the loadings (see SI Fig. 1). Previous publications have investigated water dispersion and diffusion into polypropylene (PP) films^31,32^. Shen et al. determined the presence of water molecules within the PP matrix. Even if PP has a low permeability compared to other polymers, their results have indicated molecular interactions between water molecules and the polymer matrix. The corresponding analysis of the obtained IR-ATR spectra in the present studies verified these findings via the O–H bending vibration in the range 1750–1540 cm^−1^^32^. These results indicate that residual water may be readily detected in surgical mask materials, even after a considerable drying time (here, 2 h). In Fig. 3, a minor overlap of confidence ellipses (95%) is evident, which is attributed to the spectral similarity of neat water to the H_2_O bending vibration in BSA, as it has been dissolved in aqueous solution.

**Table 1:** Calibration and classification statistics of the calculated PLS-DA models based on IR-ATR spectra.

	Analyte	RMSEC	RMSECV	R^2^ Cal	R^2^ CV
ATR	H_2_O/BSA (Num. of LVs: 2, 47,65% cum. variance captured)				
	H2O	0.2169	0.2659	0.8117	0.7192
	BSA	0.2169	0.2659	0.8117	0.7192
	BSA/VLP/unused/AAV (Num. of LVs: 3, 98,90% cum. variance captured)				
	BSA	0.0339	0.0420	0.9939	0.9906
	VLP	0.0480	0.0641	0.9877	0.9781
	unused	0.0975	0.1223	0.9493	0.9209
	AAV	0.0987	0.1245	0.9480	0.9180
ER	BSA/VLP/unused (Num. of LVs: 2, 97.17% cum. variance captured)				
	BSA	0.1326	0.1530	0.9230	0.8990
	VLP	0.0961	0.1194	0.9555	0.9315
	unused	0.1688	0.1961	0.8752	0.8327

In a next set of experiments, the classification of unused masks vs. masks exposed to VLPs, AAV, and BSA was evaluated.

The resulting PLS-DA model was built using three LVs following the optimum RMSECV value. Consequently, a RMSEC within 0.0339 and 0.0987, and a RMSECV within 0.0420 and 0.1245 was derived. For BSA, exceedingly high R^2^ values were obtained ensuring unambiguous identification (see Figure SI2A).

**Figure 4:**
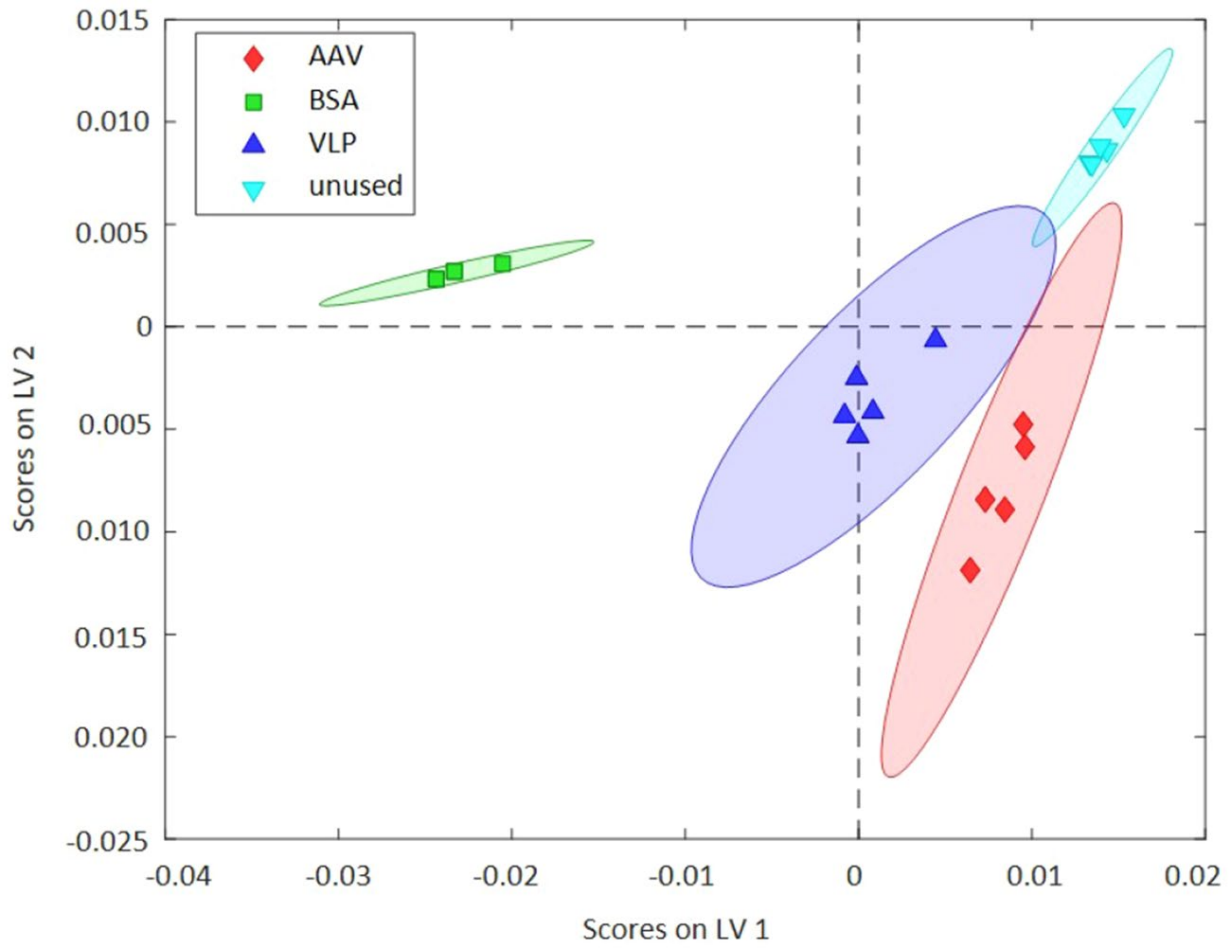
Scores plot of PLS-DA evaluation comparing four classes of face masks via IR-ATR spectroscopy. The model covers 98.90% of total variance and is based on three LVs. Unambiguous classification of differently contaminated protective face masks also vs. untreated masks is clearly evident.

As shown in Fig. 4, BSA widely separates from the VLP and AAV clusters along LV 1, which captures 77.99% of the variance. Evaluating the loadings of LV 1 in more detail, it is evident that wavelengths in the range of the water signature are apparent. These loadings indicate that structural changes of sprayed BSA at and/or within the PP matrix may be detected via direct IR-ATR spectroscopy (see SI Fig. 3). Likewise, the classification and the corresponding R^2^ values for the classes VLP and AAV indicate superior predictive accuracy ranging from 0.9180 to 0.9877, yet, minutely poorer class statistics compared to BSA (see Figure SI 2B). Evidently, the data points of these two classes are spaced wider apart within the scores plot (see Fig. 4) indicating a reduced correlation between different spots analyzed at the mask surface. In addition, it should be noted that indeed only a few microliters of each solution were deposited onto the masks, thus, some analyzed spots may also include uncontaminated mask areas. Noteworthy, a clearly evident differentiation on LV 1 compared to BSA and on LV 2 discriminating unused masks was obtained (see SI Fig. 2B). The loadings of both LV 1 and LV 2 evidence strong peaks around 1375 cm^−1^, which are attributed to the characteristic spectrum of PP masks (see Figure SI 3)^33^. These findings suggest that the sprayed-on proteins may cause minute changes of the polymer spectrum. Most importantly, the PLS-DA model facilitates an excellent differentiation of used vs. unused masks, and in addition, classifies proteins vs. virus surrogates based on rather minimal differences in IR spectral signatures. Consequently, 98.90% of the total variance across all cases studied herein may be captured via only three LVs, which confirms that indeed IR-ATR spectroscopy may extract unique spectral features associated within the molecular structure of contaminants at used protective face masks.

### IR- ER analysis of protective face masks

**Figure 5:**
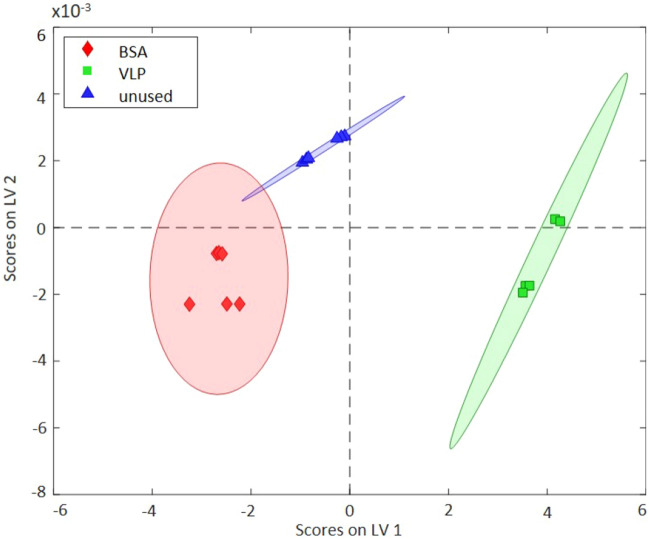
Scores plot of PLS-DA evaluation of three different classes of contaminated protective face masks analyzed via IR-ER spectroscopy. The classification results confirm unambiguous differentiation of the contaminants.

In a final set of experiments, the obtained IR-ATR results were compared to—and verified by—external reflection measurements. All data were again baseline corrected, normalized, and mean-centered as previously described. The scores plot in Fig. 5 shows that the three classes are clearly discriminated by 95% confidence ellipses based on two latent variables. These two LVs were selected covering 92.72% of the total variance. Importantly, even BSA and VLPs were unambiguously classified with R^2^ values conforming satisfying predictive accuracy (i.e., average values > 0.85). More precisely, with a high percentage of 68.55% scores along LV 1 predominantly contribute to the explanation of the overall variance, thereby indicating excellent discrimination vs. unused masks (see Figure SI 4). Noteworthy, in comparison to IR-ATR measurements, IR-ER provides an even more convenient direct measurement procedure. The considerably larger measurement spot (spot diameter approx. 12 mm in ER vs. approx. 400 µm in ATR mode, respectively) covers a more representative mask area, and the mask simply needs to be held against the aperture. Yet, IR-ATR data provided slightly more discriminatory power. Nonetheless, both methods revealed the potential of direct IR spectroscopy at protective face masks in combination with multivariate data classification routines for discriminating a variety of relevant biological species including virus surrogates. It is noteworthy, that for all three models generated within this study the sensitivity and the selectivity for the differentiation of all classes within the individual models was 1.000. Hence, also the classification error (calculated via class error = average of false positive rate and false negative rate for class, = 1—(sensitivity + specificity)/2)) is near zero (< 10E−11).

## Conclusions

In this study, it was demonstrated that IR spectroscopy combined with multivariate data analysis provides true potential for the classification of disposable protective face masks and therein trapped contaminants. Minimal spectral differences enable virus detection—and potentially discrimination—at worn protective face masks, as shown for surrogate virus examples (i.e., AAV and VLPs). Especially for health care workers and first responders these masks are part of the personal protective equipment, and thus, no additional sampling is required for testing. When compared to other testing routines, most are designed for single usage.

In this context, interest has grown in analyzing saliva as COVID-19 marker matrix. Several studies have shown that SARS-CoV-2 may be detected in full saliva at concentrations as low as approx. 1580 genome copies/mL using infrared spectroscopy. These studies also evaluated saliva-based detection for diagnostic purposes achieving sensitivities of > 90 and specificities of > 80%^34–38^. Overall, these publications clearly substantiate the claims of the present study to potentially utilize (FT)IR spectroscopy as a rapid and cost-effective, yet reliable diagnostic tool for deriving potential exposition to contagious viral material, and especially the corona virus. In contrast to the published literature, the present approach does not rely on sampling body fluids to distinguish between healthy and diseased patients^39^. Utilizing face masks as sampling objects to pre-concentrate any matter that individuals may have been exposed to is becoming increasingly relevant, yet, remains a neglected strategy especially for routine screening scenarios^40^.

Consequently, the combination of analyzing face masks with label-free read-out via IR spectroscopy and chemometric data evaluation is a unique and innovative strategy to directly detect exposition to potentially hazardous environments prior to any impacts on a person’s health. Using face masks as a readily available ‘sampling device’ offers an innovative approach for rapidly testing exposure of clinical staff toward relevant pathogens. On top of that, using IR spectroscopy in ATR or ER mode provides an affordable, portable and reusable tool for routing screening at a large scale. In this proof-of-concept study, we did not evaluate the sensitivity of SARS-CoV-2 detection in the context of other molecules contained in exhaled breath and thus on the surface of used face masks, e.g. salivary proteins or exhaled metabolites. This and differentiation of different respiratory viruses will be subject of future studies. However, given that infrared spectroscopy has been successfully used to detect SARS-CoV-2 in saliva^34,38^, which contains a plethora of bioactive molecules and proteins, we are confident that detection of viral material in breath condensate on masks will also be possible and at least as sensitive/specific as in saliva.

Together, the proposed strategy devises the first monitoring routine for respiratory viral pathogens that does not require additional sampling procedures, essentially produces no additional waste, and does not require trained personnel. Since wearing protective face masks has nowadays become routine during respiratory disease season, this approach is clearly suitable for detecting other infectious diseases of the respiratory tract as well. In a next step, the preliminary results presented in this highly actual study will be verified in both, clinical and non-clinical studies investigating extended test groups. Cross-contamination as well as exposure in real world scenarios must be evaluated in detail vs. the first insight generated herein using representative surrogate samples. In summary, the development of optical sensing technologies appears a viable strategy for virus exposure or exhalation detection that should be expanded also into other spectral regimes. Finally, using ubiquitously available used protective face masks one may convert a largely overlooked ‘sampling tool’ into a valuable dosimeter-type monitoring device that may mitigate several drawbacks of conventional rapid antigen testing routines, especially in professional application scenarios.

## Supplementary Information

Supplementary Information.
