# Supplementary material for: Towards the direct detection of viral materials at the surface of protective face masks via infrared spectroscopy
Source: Sci Rep. 2022 Feb 10;12:2309. doi: 10.1038/s41598-022-06335-z (PMC8831636; doi:10.1038/s41598-022-06335-z)
Supplement: Supplementary file 1 — Supplementary Information. [file 41598_2022_6335_MOESM1_ESM.docx]

Supporting Information

Towards the Direct Detection of Viral Materials at the Surface of Protective Face Masks via Infrared Spectroscopy

Vanessa Schorer, ^a^ Julian Haas, ^b^ Robert Stach, ^b^ Vjekoslav Kokoric, ^b^ Jan Münch, ^c^ Rüdiger Groß, ^c^ Tim Hummel, ^d, e^ Harald Sobek, ^d^ Jan Mennig, ^d^ and Boris Mizaikoff ^a, b, *^


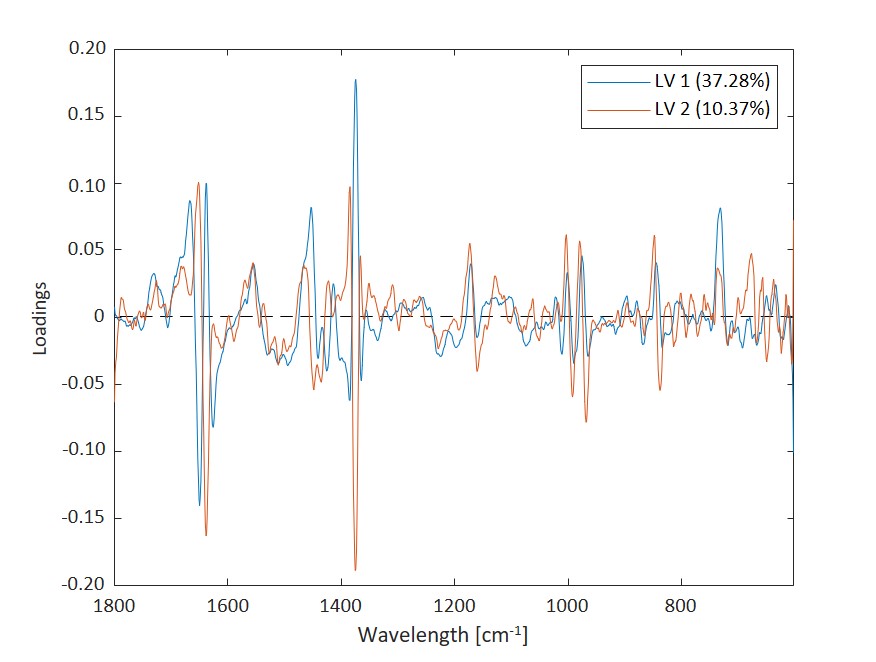


Figure SI 1 Loadings of the PLS-DA model of differently treated face masks (H_2_O and BSA). The loadings on LV 1 and LV 2 depict the characteristic H_2_O bending around 1640-1670 cm^-1^. The loadings represent the weighting of the individual variables, i.e., wavenumber as derived by the PLS-DA model based on the pre-processed spectral data.

| 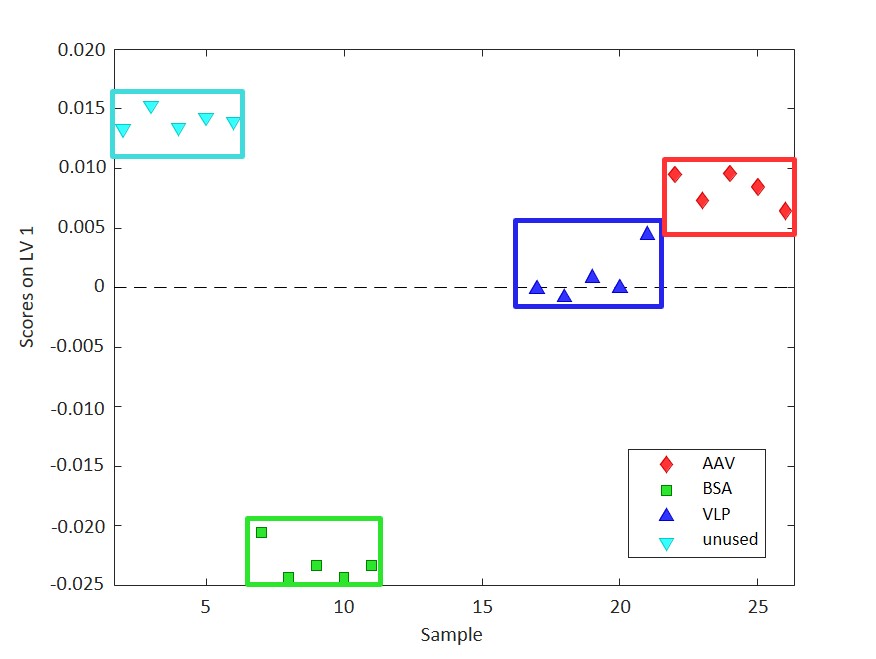A | 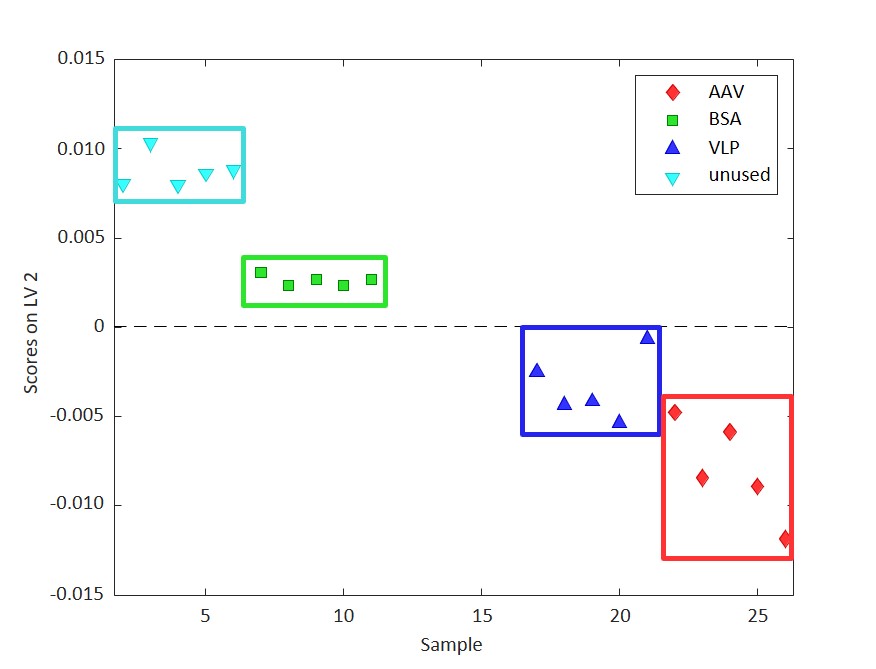B |
| --- | --- |


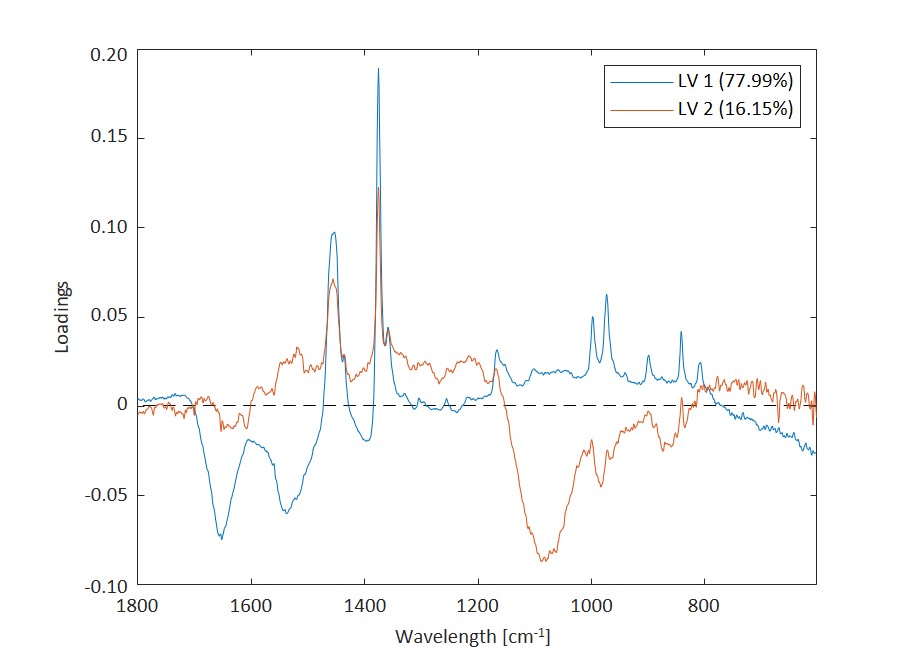
Figure SI 2 Comparison of the scores on LV 1 and LV 2 for the PLS-DA model of face masks comparing four different classes using ATR FT-IR spectral data. (A) Especially, the BSA class (green) segregates on LV 1. (B) According to the scores of LV 2 a differentiation of the classes unused/BSA versus AAV/VLP was achieved.

Figure SI 3 Loadings of LV 1 and LV 2 of the PLS-DA model evaluating different treated face masks (unused/BSA/VLP/AAV). Intensive peaks around 1375 cm^-1^ indicate the sprayed-on proteins lead to changes in polymer matrix of polypropylene. The loadings represent the weighting of the individual variables, i.e. wavenumber as derived by the PLS-DA model based on the pre-processed spectral data.

| 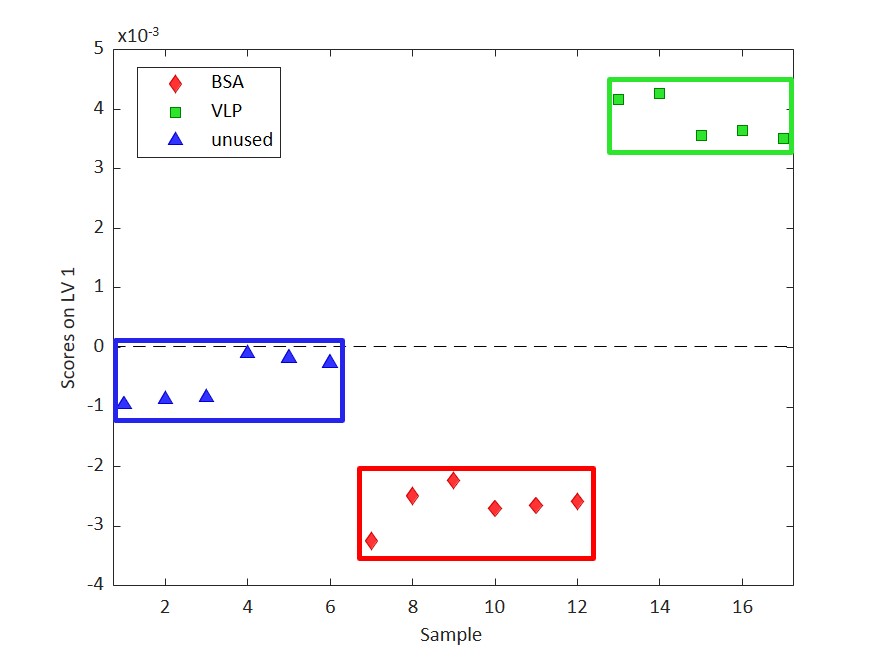A | 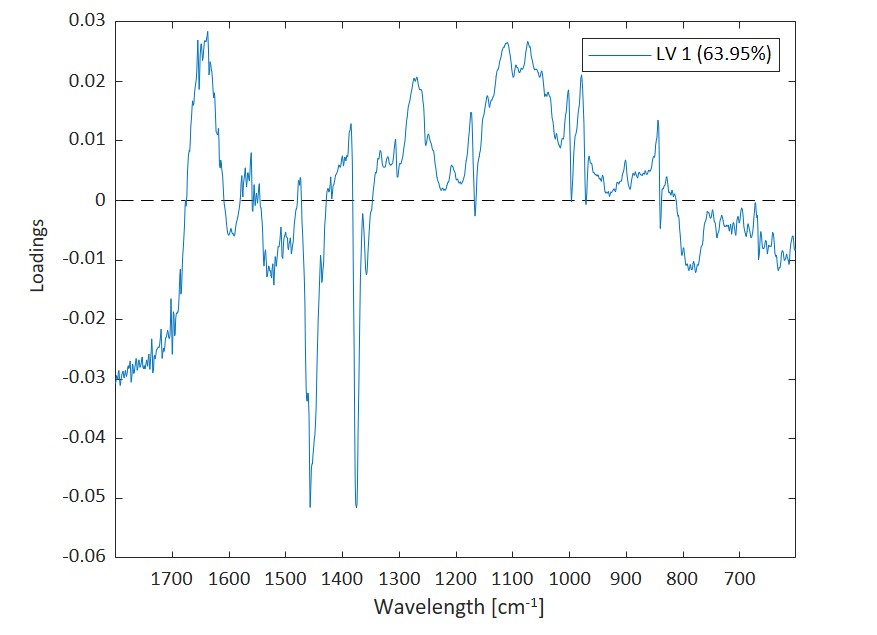B |
| --- | --- |

Figure SI 4 PLS-DA of ER FT-IR measurements analysing unused face masks with face masks on which BSA and VLPs were applied. The clear discrimination between VLPs and BSA shows that even two proteins can be classified
